# Glances at the Hospitals

**Published:** 1904-01-30

**Authors:** 


### GLANCES AT THE HOSPITALS.

### ASHTON-UNDER-LYNE INFIRMARY.

The total expenditure was £5,272, and the year closed with an adverse balance of £1,414. Many alterations have been carried out. The operating theatre has been enlarged and improved, a new heating apparatus has been fitted up, electric fans have been put in, and new baths have been added. Exclusive of the operating theatre these have cost a sum of £2,353, and the theatre has cost £620. The daily average of beds occupied was 52, and the cost per bed occupied was £62 10s. The average stay in the hospital was 31 days. The medical and surgical statistics are clearly arranged so far as they go, but there is neither a table of operations nor a table of anæsthetics used, although 228 operations were performed among the in-patients.

### ADDENBROOKE'S HOSPITAL, CAMBRIDGE.

The total ordinary income was £7,347, and the total ordinary expenditure was £9,229 The deficit of £2,100 was carried to the capital account. The total number of in-patients was 1,533, and the total number of out-patients 5,403. The number of surgical operations performed was 892. A medical school is attached to the hospital, and it is recognised by the various examining boards of Great Britain. This makes it more remarkable that no medical statistics are given in the report. The training school for nurses dates from 1877, and a three-years' course is in operation. Probationers pay £30 for the three years training.

### WOLVERHAMPTON HOSPITAL FOR WOMEN.

The income was £1,713, and the expenditure £2,123. A balance of £306 was due to the Bank. It is proposed to build a new hospital, and for this purpose a sum of £3,784

has already been received. It is to be hoped that the public will cheerfully and substantially respond to the manager's appeal for funds, as there cannot be a doubt of the advisableness of the step contemplated. During the year 223 operations were performed with a mortality of 1·3 per cent. One hundred and two of the operations were abdominal sections and these showed a mortality of 2 9 per cent. The tables show the date of operation, age of patient, disease, nature of operation, and the result. There is also a mortality table with a column for remarks on the post-mortem examination. Beyond a table of anæsthetics we do not miss anything as regards medical statistics, but it would be desirable that future reports should state the number of patients in the hospital at the beginning and end of the year, the number admitted, the stay in the wards, the cost per head, and the cost per bed occupied.

